# Astaxanthin Confers a Significant Attenuation of Hippocampal Neuronal Loss Induced by Severe Ischemia-Reperfusion Injury in Gerbils by Reducing Oxidative Stress

**DOI:** 10.3390/md20040267

**Published:** 2022-04-14

**Authors:** Joon Ha Park, Tae-Kyeong Lee, Dae Won Kim, Ji Hyeon Ahn, Choong-Hyun Lee, Jong-Dai Kim, Myoung Cheol Shin, Jun Hwi Cho, Jae-Chul Lee, Moo-Ho Won, Soo Young Choi

**Affiliations:** 1 Department of Anatomy, College of Korean Medicine, Dongguk University, Gyeongju 38066, Korea; jh-park@dongguk.ac.kr; 2 Department of Food Science and Nutrition, Hallym University, Chuncheon 24252, Korea; tk_lee@hallym.ac.kr; 3 Department of Biochemistry and Molecular Biology, Research Institute of Oral Sciences, College of Dentistry, Gangnung-Wonju National University, Gangneung 25457, Korea; kimdw@gwnu.ac.kr; 4 Department of Physical Therapy, College of Health Science, Youngsan University, Yangsan 50510, Korea; jh-ahn@ysu.ac.kr; 5 Department of Pharmacy, College of Pharmacy, Dankook University, Cheonan 31116, Korea; anaphy@dankook.ac.kr; 6 Division of Food Biotechnology, School of Biotechnology, Kangwon National University, Chuncheon 24341, Korea; jongdai@kangwon.ac.kr; 7 Department of Emergency Medicine, Kangwon National University Hospital, School of Medicine, Kangwon National University, Chuncheon 24289, Korea; dr10126@naver.com (M.C.S.); cjhemd@kangwon.ac.kr (J.H.C.); 8 Department of Neurobiology, School of Medicine, Kangwon National University, Chuncheon 24341, Korea; anajclee@kangwon.ac.kr; 9 Department of Biomedical Science and Research Institute for Bioscience and Biotechnology, Hallym University, Chuncheon 24252, Korea

**Keywords:** antioxidant enzymes, DNA damage, ischemia and reperfusion, lipid peroxidation, lipid-soluble carotenoid, neuroprotection

## Abstract

Astaxanthin is a powerful biological antioxidant and is naturally generated in a great variety of living organisms. Some studies have demonstrated the neuroprotective effects of ATX against ischemic brain injury in experimental animals. However, it is still unknown whether astaxanthin displays neuroprotective effects against severe ischemic brain injury induced by longer (severe) transient ischemia in the forebrain. The purpose of this study was to evaluate the neuroprotective effects of astaxanthin and its antioxidant activity in the hippocampus of gerbils subjected to 15-min transient forebrain ischemia, which led to the massive loss (death) of pyramidal cells located in hippocampal cornu Ammonis 1-3 (CA1-3) subfields. Astaxanthin (100 mg/kg) was administered once daily for three days before the induction of transient ischemia. Treatment with astaxanthin significantly attenuated the ischemia-induced loss of pyramidal cells in CA1-3. In addition, treatment with astaxanthin significantly reduced ischemia-induced oxidative DNA damage and lipid peroxidation in CA1-3 pyramidal cells. Moreover, the expression of the antioxidant enzymes superoxide dismutase (SOD1 and SOD2) in CA1-3 pyramidal cells were gradually and significantly reduced after ischemia. However, in astaxanthin-treated gerbils, the expression of SOD1 and SOD2 was significantly high compared to in-vehicle-treated gerbils before and after ischemia induction. Collectively, these findings indicate that pretreatment with astaxanthin could attenuate severe ischemic brain injury induced by 15-min transient forebrain ischemia, which may be closely associated with the decrease in oxidative stress due to astaxanthin pretreatment.

## 1. Introduction

Brains are very sensitive and vulnerable to disruption of the blood supply. Transient cerebral ischemia is a medical emergency that results from the temporary cessation of the blood supply to the brain, which results in irrecoverable neuronal damage in various brain regions [1,2]. Compared to other brain regions, the hippocampus is the region most vulnerable to ischemic injury, where neuronal loss (death) in the hippocampal CA1 occurs a few days after five minutes of transient forebrain ischemia [3,4]. The extent and time of neuronal death in the hippocampus are closely correlated with the duration of transient ischemia [5,6,7]. The diverse mechanisms of hippocampal neuronal death following transient ischemia (ischemia and reperfusion, IR) have been addressed, and, among the mechanisms, oxidative stress caused by the overgeneration of reactive oxygen species (ROS) following IR is well understood [8,9]. Hence, antioxidant strategies to improve cellular defenses against oxidative stress in IR injury have been proposed as useful therapeutic approaches [10,11]

Astaxanthin (ATX), a lipid-soluble carotenoid, is a powerful biological antioxidant and exists widely in natural organisms, including marine organisms, green plants, and microorganisms [12]. It exerts a broad spectrum of pharmacological effects, such as anticoagulant, anti-inflammatory, and antioxidant activities with the characteristic of low toxicity [13,14,15]. ATX has been reported to have higher antioxidant activity than a range of carotenoids including α-carotene, β-carotene, lycopene, and lutein [16]. In addition, ATX can cross the blood-brain barrier (BBB) and accumulate in the brain, where it can potentially provide a beneficial effect [17]. Due to these advantages, ATX has been regarded as a promising therapeutic agent for neurological diseases [18].

ATX has been demonstrated to exert neuroprotective effects via antioxidant activity in experimental animal models of cerebral ischemia. Zhang et al. (2020) showed that pretreatment with ATX protected the SH-SY5Y neuronal cells from oxygen and glucose deprivation-induced oxidative damage [19], and Pan et al. (2017) showed that pretreatment with ATX significantly reduced infarction volume in rat brains after transient focal brain ischemia, via activation of the antioxidant defense pathway [20]. Pretreatment with ATX was also reported to protect hippocampal neurons from repeated IR-induced brain injury in mice, suggesting that the effect might be related to the alleviation of oxidative stress [21]. Furthermore, it has been demonstrated that treatment with ATX significantly reduces brain injury following transient focal cerebral ischemia induced by middle cerebral artery occlusion in rats via antioxidant efficacy [22,23]. In addition, treatment with ATX ameliorates neuronal loss in the spinal cord following IR in rats via activation of the PI3K/Akt/GSK-3 beta pathway [24].

However, to the best of our knowledge, the neuroprotective effects of ATX against severe IR injury in the forebrain induced by a long period of transient ischemia have not been explored yet, and it is unclear whether ATX can attenuate severe IR injury in the forebrain by antioxidative activity. Thus, the aim of this experiment was to investigate the neuroprotective effects of ATX in a gerbil model of 15-min transient forebrain ischemia which results in massive neuronal loss (death) in the hippocampal CA1-3 [7,25]. In addition, we examined whether the neuroprotective effects were closely associated with the potent antioxidant activity of ATX.

## 2. Results

### 2.1. Neuroprotection by ATX

#### 2.1.1. Findings by Nissl Staining

**Figure 1:**
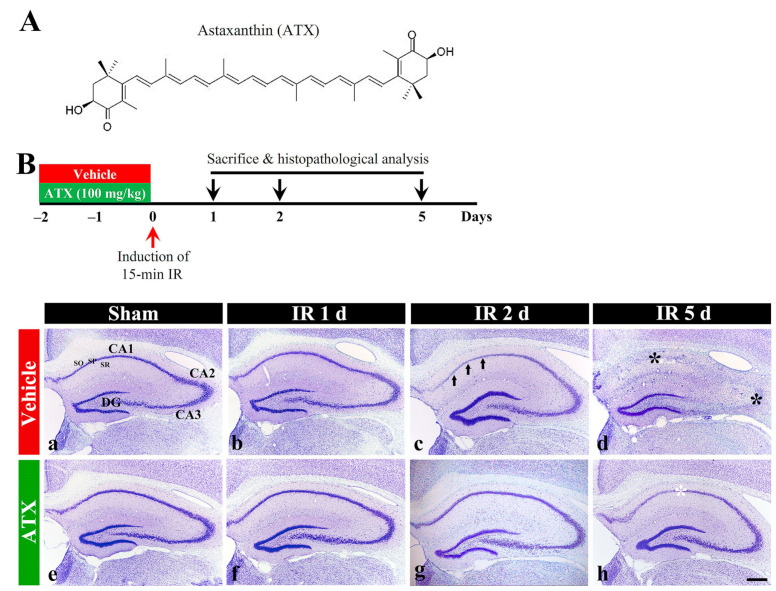
(**A**) The chemical structure of ATX and experimental procedure. ATX is dissolved in saline and orally administered once a day for three days before IR induction. The gerbils are sacrificed on day 1, 2 and 5 after IR; (**B**) Representative photographs of Nissl staining in the hippocampus (CA1-3) of the vehicle-treated (upper low) and ATX-treated (lower low) groups in sham (**a**,**e**), and on day 1 (**b**,**f**), day 2 (**c**,**g**), and day 5 (**d**,**h**) after IR. In the vehicle-IR group, CV stainability in the SP of CA1 (arrows in (**c**)) is pale at two days after IR, and, at five days after IR, very pale CV stainability is shown in the SP of CA1-3 (black asterisks in (**d**)). In the ATX-IR group, pale CV stainability is shown only in the SP of CA1 (white asterisk in (**h**)) at five days after IR. DG, dentate gyrus; SO, stratum oriens; SR, stratum radiatum. Scale bar = 400 μm.

In the vehicle-sham group, intact cells stained with cresyl violet (CV), which have intact Nissl substance (aggregation of rough endoplasmic reticulum) in the cytoplasm, were distinctly observed in the stratum pyramidale (SP) in the hippocampal proper (CA1-CA3) (Figure 1(Ba)). In the vehicle-IR group, CV stainability at one day after IR was not significantly altered compared with the vehicle-sham group (Figure 1(Bb)). However, at two days after IR, CV stainability was decreased in the SP of CA1 (Figure 1(Bc)), and, at five days after IR, a significant loss of CV stainability was observed in the SP of CA1-3 (Figure 1(Bd)). In the ATX-sham group, CV stainability in the SP of CA1-3 was not different from that of the vehicle-sham group (Figure 1(Be)). In the ATX-IR group, a significant change in CV stainability was not observed until two days after IR (Figure 1(Bf,Bg)), and, at five days after IR, CV stainability was weak in the SP of CA1 alone (Figure 1(Bh)). This finding means that severe IR damages Nissl substance, and ATX could protect it from IR injury.

#### 2.1.2. Findings by Neuronal Nuclei (NeuN, a Marker for Mature Neurons) Immunohistochemistry

**Figure 2:**
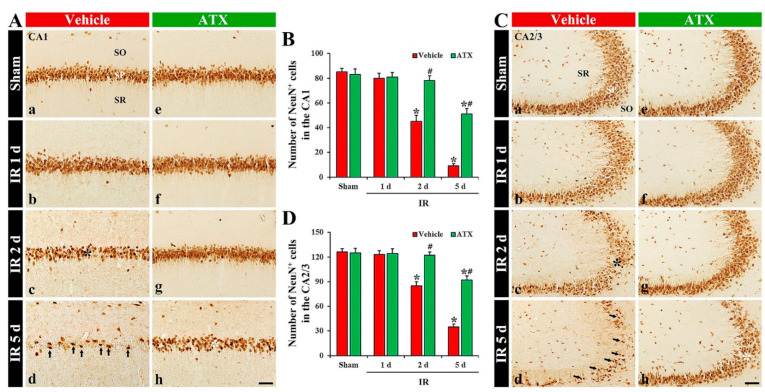
(**A**,**C**) Representative photographs of NeuN immunohistochemistry in CA1 (**A**) and CA2/3 (**C**) of the vehicle-treated (left column) and ATX-treated (right column) groups in sham (**Aa**,**Ca**,**Ae**,**Ce**), and on day 1 (**Ab**,**Cb**,**Af**,**Cf**), day 2 (**Ac**,**Cc**,**Ag**,**Cg**), and day 5 (**Ad**,**Cd**,**Ah**,**Ch**) after IR. In the vehicle-IR group, a loss of NeuN-cells (black asterisks in (**Ac**,**Cc**)) in the SP of CA1-3 is observed on day 2 after IR. At five days after IR, NeuN-pyramidal cells of CA1-3 (arrows in (**Ad**,**Cd**)) are markedly decreased; however, in the ATX-IR group, many NeuN-pyramidal cells (white asterisks in (**Ah**,**Ch**)) are shown. Scale bar = 60 µm; (**B**,**D**) Mean numbers of NeuN-pyramidal cells in CA1 (**B**) and CA2/3 (**D**). The bars indicate the means ± SEM (*n* = 7, respectively; * *p* < 0.05 vs. corresponding sham group, ^#^ *p* < 0.05 vs. vehicle-IR group).

In the vehicle-sham group, cells in the SP (named pyramidal cells or neurons) of CA1-3 were shown to have clear NeuN immunoreactivity (Figure 2(Aa,Ca)). In the vehicle-IR group, NeuN-immunostained cells (NeuN-pyramidal cells) of CA1-3 at one day after IR were not altered in morphology and numbers (Figure 2(Ab,B,Cb,D)). However, NeuN-pyramidal cells of CA1-3 were significantly decreased (67% of the vehicle-sham group) in numbers at two days after IR (Figure 2(Ac,B,Cc,D)), and, at five days after IR, a massive loss (27% of the vehicle-sham group) of NeuN-pyramidal cells of CA1-3 were observed, showing that the number in CA1 and CA2/3 was 9 and 35 cells/300 μm^2^, respectively (Figure 2(Ad,B,Cd,D)). In the ATX-sham group, NeuN-pyramidal cells in CA1-3 were not different in numbers and morphology from the vehicle-sham group (Figure 2(Ae,B,Ce,D)). In the ATX-IR group, NeuN-pyramidal cells were not significantly changed until two days after IR (Figure 2(Ae,Af,B,Cf,Cg,D)), and, at five days after IR, NeuN-pyramidal cells of CA1-3 were decreased in numbers, but the number in CA1 and CA2/3 was higher (603% and 268% of the vehicle-IR group) than the vehicle-IR group, showing that the number in CA1 and CA2/3 was 51 and 92 cells/300 μm^2^, respectively (Figure 2(Ah,B,Ch,D)). This finding means that severe IR damages the NeuN protein located in the nucleus, and ATX could protect it from IR injury.

#### 2.1.3. Findings by Fluoro-Jade B (FJB) Histofluorescence

**Figure 3:**
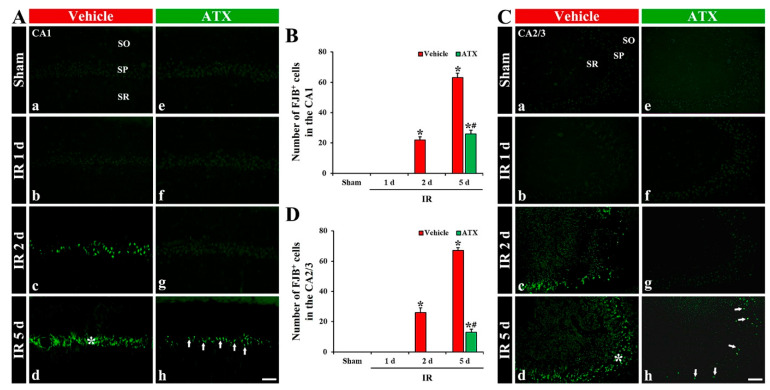
(**A**,**C**) Representative photographs of FJB staining in CA1 (**A**) and CA2/3 (**C**) of the vehicle-treated (left column) and ATX-treated (right column) groups in sham (**Aa**,**Ca**,**Ae**,**Ce**), and on day 1 (**Ab**,**Cb**,**Af**,**Cf**), day 2 (**Ac**,**Cc**,**Ag**,**Cg**), and day 5 (**Ad**,**Cd**,**Ah**,**Ch**) after IR. In the vehicle-IR group, FJB-pyramidal cells are detected in the SP of CA1-3 from two days after IR, and, at five days after IR, FJB-pyramidal cells (white asterisks in (**Ad**,**Cd**)) are significantly increased. However, in the ATX-IR group, decreased FJB-pyramidal cells (arrows in (**Ah**,**Ch**)) are observed in CA1-3 only at five days after IR. Scale bar = 60 µm; (**B**,**D**) Mean numbers of FJB- pyramidal cells in CA1 (**B**) and CA2/3 (**D**). The bars indicate the means ± SEM (*n* = 7, respectively; * *p* < 0.05 vs. corresponding sham group, ^#^ *p* < 0.05 vs. vehicle-IR group).

In the vehicle-sham and ATX-sham groups, no FJB-stained cells (FJB-cells), which are degenerating or dead cells, were detected in CA1-3 (Figure 3(Aa,Ae,Ca,Ce)). In the vehicle-IR group, FJB-pyramidal cells in CA1-3 were observed from two days after IR (Figure 3(Ab,Ac,B,Cb,Cc,D)), and FJB-pyramidal cells of CA1-3 were dramatically increased at five days after IR; the number of FJB-pyramidal cells in CA1 and CA2/3 was 63 and 67 cells/300 μm^2^, respectively (Figure 3(Ad,B,Cd,D)). In the ATX-IR group, FJB-pyramidal cells in CA1-3 were not found until two days after IR (Figure 3(Af,Ag,B,Cf,Cg,D)). At five days after IR, many FJB-pyramidal cells were detected in CA1 and CA2/3 (26 and 13 cells/300 μm^2^, respectively), but the number was significantly low (41% in CA1 and 19% in CA2/3 of vehicle-IR group) when compared with the vehicle-IR group (Figure 3(Ah,B,Ch,D)). This finding means that the pyramidal cells in CA1-3 were lost (dead) after severe IR, and ATX could attenuate the death of pyramidal cells induced by severe IR.

### 2.2. Attenuation of Oxidative DNA Damage and Lipid Peroxidation by ATX

#### 2.2.1. 8-Hydroxydeoxyguanosine (8OHdG a Marker of Oxidative DNA Damage) Immunoreactivity

**Figure 4:**
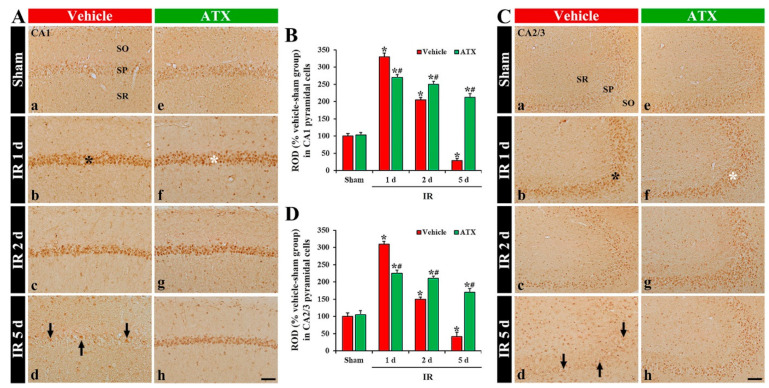
(**A**,**C**) Representative photographs of 8OHdG immunohistochemistry in CA1 (**A**) and CA2/3 (**C**) of the vehicle-treated (left column) and ATX-treated (right column) groups in sham (**Aa**,**Ca**,**Ae**,**Ce**), and on day 1 (**Ab**,**Cb**,**Af**,**Cf**), day 2 (**Ac**,**Cc**,**Ag**,**Cg**), and day 5 (**Ad**,**Cd**,**Ah**,**Ch**) after IR. In the vehicle-IR group, 8OHdG immunoreactivity in CA1-3 pyramidal cells (black asterisks in (**Ab**,**Cb**)) is significantly increased at one day after IR. However, in the ATX-IR group, 8OHdG immunoreactivity in CA1-3 pyramidal cells (white asterisks in (**Af**,**Cf**)) at one day after IR is significantly low when compared with the vehicle-IR group. Note that, at five days after IR, 8OHdG immunoreactivity is very weak (arrows in (**Ad**,**Cd**)) in the vehicle-IR group due to death of pyramidal cells. Scale bar = 60 µm; (**B**,**D**) ROD of 8OHdG immunoreactivity in CA1 (**B**) and CA2/3 (**D**). The bars indicate the means ± SEM (*n* = 7, respectively; * *p* < 0.05 vs. corresponding sham group, ^#^ *p* < 0.05 vs. vehicle-IR group).

In the vehicle-sham group, 8OHdG immunoreactivity was weakly shown in the intact pyramidal cells of CA1-3 (Figure 4(Aa,Ca)). In the vehicle-IR group, 8OHdG immunoreactivity in the CA1 and 2/3 pyramidal cells was significantly increased (331% and 310% of the vehicle-sham group, respectively) at one day after IR (Figure 4(Ab,B,Cb,D)). At two days after IR, 8OHdG immunoreactivity in the CA1-3 pyramidal cells was decreased; at this point in time, many of the CA1-3 pyramidal cells were lost (dead) (Figure 4(Ac,B,Cc,D)). At five days after IR, 8OHdG immunoreactivity in the CA1-3 pyramidal cells was more significantly decreased: at this time, an extensive loss of the CA1-3 pyramidal cells occurred (Figure 4(Ad,B,Cd,D)). In the ATX-sham group, 8OHdG immunoreactivity in the pyramidal cells of CA1-3 was not different from that shown in the vehicle-sham group (Figure 4(Ae,B,Ce,D)). In the ATX-IR group, 8OHdG immunoreactivity in the CA1 and CA2/3 pyramidal cells was increased (278% and 226% of the ATX-sham group, respectively) at one day after IR, but the immunoreactivity was significantly lower (82% and 72% of the vehicle-IR group, respectively) than that in the vehicle-IR group (Figure 4(Af,B,Cf,D)). Thereafter, 8OHdG immunoreactivity in the CA1-3 pyramidal cells was slightly and gradually decreased with time (Figure 4(Ag,Ah,B,Cg,Ch,D)). This finding means that damage of DNA in the pyramidal cells of CA1-3 was significant at one day after severe IR, and ATX could attenuate the DNA damage induced by severe IR.

#### 2.2.2. 4-Hydroxy-2-Nonenal (4HNE, a Marker for Lipid Peroxidation) Immunoreactivity

**Figure 5:**
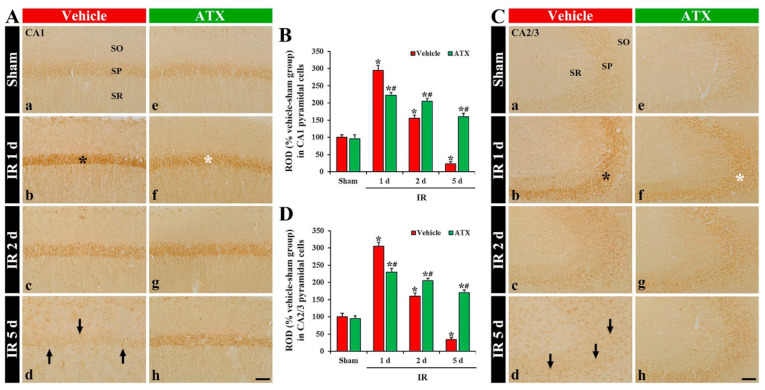
(**A**,**C**) Representative photographs of 4HNE immunohistochemistry in CA1 (**A**) and CA2/3 (**C**) of the vehicle-treated (left column) and ATX-treated (right column) groups in sham (**Aa**,**Ca**,**Ae**,**Ce**), and on day 1 (**Ab**,**Cb**,**Af**,**Cf**), day 2 (**Ac**,**Cc**,**Ag**,**Cg**), and day 5 (**Ad**,**Cd**,**Ah**,**Ch**) after IR. Black asterisks (in (**Ab**,**Cb**)) indicate that, in the vehicle-IR group, 4HNE immunoreactivity is significantly increased in CA1-3 pyramidal cells at one day after IR. However, in the ATX-IR group, 4HNE immunoreactivity in CA1-3 pyramidal cells (white asterisks in (**Af**,**Cf**)) at 1 day after IR is significantly low when compared with in the vehicle-IR group. Note that, at five days after IR, 4HNE immunoreactivity is very weak (arrows in Ad and Cd) in the vehicle-IR group due to death of pyramidal cells. Scale bar = 60 µm; (**B**,**D**) ROD of 4HNE immunoreactivity in CA1 (**B**) and CA2/3 (**D**). The bars indicate the means ± SEM (*n* = 7, respectively; * *p* < 0.05 vs. corresponding sham group, ^#^ *p* < 0.05 vs. vehicle-IR group).

In the vehicle-sham group, 4HNE immunoreactivity was weakly found in the intact CA1-3 pyramidal cells (Figure 5(Aa,Ca)). In the vehicle-IR group, 4HNE immunoreactivity in the pyramidal cells of CA1 and CA2/3 was significantly increased (296% and 305% of the vehicle-IR group, respectively) at one day after IR (Figure 5(Ab,B,Cb,D)). At two days after IR, 4HNE immunoreactivity in the CA1-3 pyramidal cells was decreased compared to that shown in the vehicle-IR group at one day after IR; at this time, many of the CA1-3 pyramidal cells died (Figure 5(Ac,B,Cc,D)). At five days after IR, 4HNE immunoreactivity was very low due to a massive loss of the CA1-3 pyramidal cells (Figure 5(Ad,B,C,D)). In the ATX-sham group, 4HNE immunoreactivity in the CA1-3 pyramidal cells was similar to the vehicle-sham group (Figure 5(Ae,B,Ce,D)). In the ATX-IR group, 4HNE immunoreactivity in CA1 and CA2/3 pyramidal cells was also increased (214% and 221% of ATX-sham group, respectively) at one day after IR, but the immunoreactivity was significantly lower than that in the vehicle-IR group (Figure 5(Af,B,Cf,D)). Thereafter, 4HNE immunoreactivity in the CA1-3 pyramidal cells was gradually and slightly decreased with time due to the attenuation of IR-induced neuronal loss (Figure 5(Ag,Ah,B,Cg,Ch,D)). This finding means that lipid peroxidation in the pyramidal cells of CA1-3 was significantly increased at one day after severe IR, and ATX could attenuate the lipid peroxidation induced by severe IR.

### 2.3. Increase of SOD1 and SOD2 Expressions by ATX

#### 2.3.1. SOD1 Immunoreactivity

**Figure 6:**
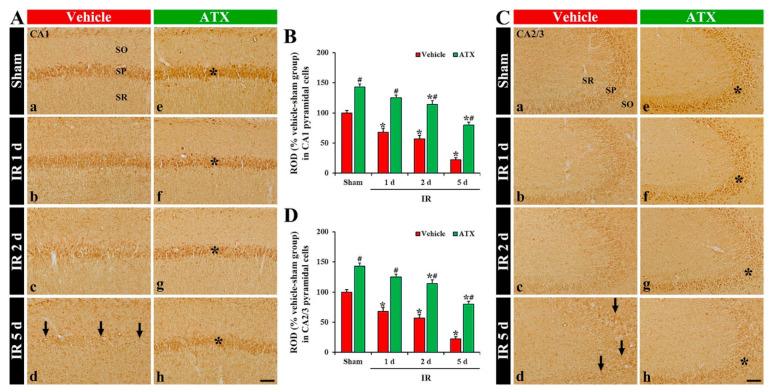
(**A**,**C**) Representative photographs of SOD1 immunohistochemistry in CA1 (**A**) and CA2/3 (**C**) of the vehicle-treated (left column) and ATX-treated (right column) groups in sham (**Aa**,**Ca**,**Ae**,**Ce**), and on day 1 (**Ab**,**Cb**,**Af**,**Cf**), day 2 (**Ac**,**Cc**,**Ag**,**Cg**), and day 5 (**Ad**,**Cd**,**Ah**,**Ch**) after IR. In the vehicle-IR group, SOD1 immunoreactivity in CA1-3 pyramidal cells is decreased with time after IR, and very low (arrows in (**Ad**,**Cd**)) on day 5 after IR. In the ATX-sham group, SOD1 immunoreactivity in CA1-3 pyramidal cells is significantly higher (asterisk in (**Ae**,**Ce**)) than in the vehicle-sham group. In the ATX-IR group, SOD1 immunoreactivity in CA1-3 pyramidal cells is decreased with time, but the immunoreactivity is significantly high (asterisks in (**Af**–**Ah**,**Cf**–**Ch**)) as compared with the vehicle-IR group. Scale bar = 60 µm; (**B**,**D**) ROD of SOD1 immunoreactivity in CA1 (**B**) and CA2/3 (**D**). The bars indicate the means ± SEM (*n* = 7, respectively; * *p* < 0.05 vs. corresponding sham group, ^#^ *p* < 0.05 vs. vehicle-IR group).

SOD1 immunoreactivity, in the vehicle-sham group, was shown in the pyramidal cells of CA1 and CA2/3 (Figure 6(Aa,Ca)). In the vehicle-IR group, SOD1 immunoreactivity in the CA1 and CA2/3 pyramidal cells was significantly decreased (69% and 71% of vehicle-sham group, respectively) on day 1 after IR (Figure 6(Ab,B,Cb,D)). On day 2 after IR, SOD1 immunoreactivity in the CA1-3 pyramidal cells was further decreased (Figure 6(Ac,B,Cc,D)), and, on day 5 after IR, SOD1 immunoreactivity was very low (56% and 21% of the vehicle-sham group, respectively) (Figure 6(Ad,B,Cd,D)) due to an extensive loss of CA1-3 pyramidal cells. In the ATX-sham group, SOD1 immunoreactivity in the CA1 and CA2/3 pyramidal cells was significantly higher (144% and 155% of the vehicle-sham group, respectively) than the vehicle-sham group (Figure 6(Ae,B,Ce,D)). In the ATX-IR group, SOD1 immunoreactivity in CA1 and CA2/3 pyramidal cells was also decreased with time, but each immunoreactivity was significantly high (on day 5 after IR, 378% and 382% of the vehicle-IR group, respectively) when compared with the ATX-IP group (Figure 6(Af–Ah,B,Cf–Ch,D)). This finding means that SOD1 in the CA1-3 pyramidal cells was significantly decreased with time after severe IR, and ATX could attenuate the decrease of SOD1 induced by severe IR.

#### 2.3.2. SOD2 Immunoreactivity

**Figure 7:**
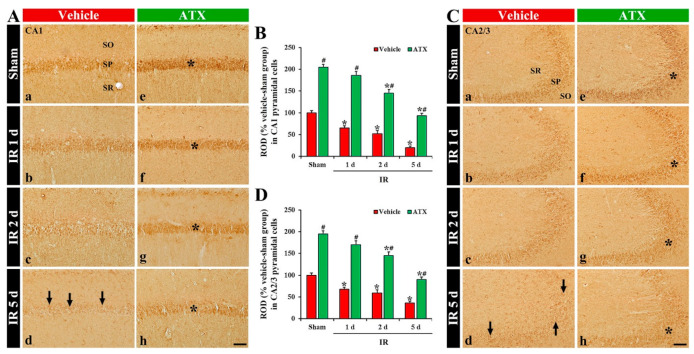
(**A**,**C**) Representative photographs of SOD2 immunohistochemistry in CA1 (**A**) and CA2/3 (**C**) of the vehicle-treated (left column) and ATX-treated (right column) groups in sham (**Aa**,**Ca**,**Ae**,**Ce**), and on day 1 (**Ab**,**Cb**,**Af**,**Cf**), day 2 (**Ac**,**Cc**,**Ag**,**Cg**), and day 5 (**Ad**,**Cd**,**Ah**,**Ch**) after IR. SOD1 immunoreactivity, in the vehicle-IR group, is decreased in CA1-3 pyramidal cells with time after IR, and very low (arrows in (**Ad**,**Cd**)) at five days after IR. In the ATX-sham group, SOD1 immunoreactivity in CA1-3 pyramidal cells is significantly higher (asterisk in (**Ae**,**Ce**)) than the vehicle-sham group. SOD1 immunoreactivity in CA1-3 pyramidal cells of the ATX-IR group is decreased with time, but the immunoreactivity is significantly high (asterisks in (**Af**–**Ah**,**Cf**–**Ch**)) when compared with the vehicle-IR group. Scale bar = 60 µm; (**B**,**D**) ROD of SOD2 immunoreactivity in CA1 (**B**) and CA2/3 (**D**). The bars indicate the means ± SEM (*n* = 7, respectively; * *p* < 0.05 vs. corresponding sham group, ^#^ *p* < 0.05 vs. vehicle-IR group).

SOD2 immunoreactivity, in the vehicle-sham group, was mainly shown in the CA1 and CA2/3 pyramidal cells (Figure 7(Aa,Ca)). In the vehicle-IR group, a significant decrease of SOD2 immunoreactivity in the CA1 and CA2/3 pyramidal cells (65% and 68% of the vehicle-sham group, respectively) was observed at one day after IR (Figure 7(Ab,B,Cb,D)). At two days after IR, SOD2 immunoreactivity in the CA1-3 pyramidal cells was more decreased (Figure 7(Ac,B,Cc,D)), and, at five days after IR, SOD2 immunoreactivity in the CA1-3 pyramidal cells was very low (51% and 18% of the vehicle-sham group, respectively) due to an extensive loss of pyramidal cells (Figure 7(Ad,B,Cd,D)). In the ATX-sham group, SOD2 immunoreactivity in the CA1 and CA2/3 pyramidal cells was significantly higher (205% and 196% of the vehicle-sham group, respectively) when compared with the vehicle-sham group (Figure 7(Ae,B,Ce,D)). In the ATX-IR group, SOD2 immunoreactivity in the CA1-3 pyramidal cells was also decreased with time, but each immunoreactivity was significantly high when compared with the vehicle-IR group (Figure 7(Af–Ah,B,Cf–Ch,D)). This finding means that SOD2 in CA1-3 pyramidal cells was also significantly decreased with time after severe IR, and ATX could attenuate the decrease of SOD2 induced by severe IR.

## 3. Discussion

The degree of hippocampal neuronal death following IR injury varies according to the ischemic duration. It has been demonstrated that the selective death of pyramidal cells in hippocampal CA1 occurs four to five days after five minutes of transient ischemia in the forebrain of gerbils [4,26]. We reported that the death of pyramidal cells in CA1-3 of gerbils subjected to 15 min of transient ischemia was observed from two days post-ischemia, indicating that a longer period of transient ischemia could evoke the acceleration and aggravation of pyramidal cell death in the hippocampus [7,25]. In this study, we examined the neuroprotective effects of ATX in CA1-3 of gerbils subjected to transient ischemia for 15 min using Nissl staining, NeuN immunohistochemistry, and FJB histofluorescence staining, and found that pretreatment with ATX significantly alleviated ischemia-induced pyramidal cell death in CA1-3. We believe that this finding was the first showing that ATX elicited strong neuroprotection against severe IR injury in the gerbil hippocampus. In rats, global cerebral ischemia for 10 min induced pyramidal cell death in CA1 alone, which might be a phenomenon of mild IR injury in the hippocampus compared to severe IR injury in the hippocampus, and the administration of ATX significantly reduced the selective death [27]. To the best of our knowledge, it is impossible to develop a rat model of severe IR injury in the hippocampus by occluding four vessels (two common carotid arteries and two vertebral arteries) for 15 min or more because rats subjected to this ischemic injury die a few days later due to failure of the brainstem which receives arterial blood supply from the vertebral arteries [28,29]. However, as described in the Materials and Methods Section, a gerbil model of severe IR injury in the hippocampus can be developed, because gerbils with severe IR injury induced by the ligation of two common carotid arteries (not two vertebral arteries) survive a long time without brainstem failure [6,7]. Regarding the anatomy of the circle of Willis in rats and gerbils, rats have a perfect circle of Willis, whereas gerbils have an incomplete circle of Willis so that there is a lack of posterior communicating arteries [7,30]. These anatomical characteristics helped us to develop a gerbil model of severe IR injury in the forebrain. Many years ago, Jarrott and Dommer (1980) reported that a mortality rate of 100% was obtained if the occlusion of bilateral common carotid arteries was maintained for 60 min in gerbils weighing 45–55 g [31].

Oxidative stress is a phenomenon induced by an imbalance between the production and accumulation of ROS in cells and/or tissues and the ability of a system to detoxify the reactive products [32]. When the production of ROS increases, harmful effects are exerted on important structures in cells such as proteins, lipids, and nucleic acids [33]. In ischemic insults to the brain, oxidative stress has also been considered to be a major factor to overcome following ischemic brain injury. Many preclinical studies have demonstrated that oxidative stress following ROS accumulation during IR irreversibly damaged cellular biomolecules, including lipids, proteins, and DNA, and led to neuronal death [8,10]. In particular, early during IR, due to an elevated oxygen supply, large quantities of oxygen radicals increase rapidly in the ischemic lesions and DNA damage occurs [34]. Lipid peroxidation can be described as a process under which oxidants including ROS attack lipids containing carbon–carbon double bond(s) [35]. In this respect, various substances reducing oxidative stress have been demonstrated to attenuate ischemic brain injury following IR injury [36,37,38]. In this study, the immunoreactivity of 8OHdG (an oxidative DNA adduct) in CA1-3 pyramidal cells was significantly enhanced on day 1 after IR (before the time of IR-induced loss of CA1-3 pyramidal cells), but it was significantly reduced by ATX treatment. Resveratrol, which is a naturally occurring polyphenolic compound and elicits a variety of biological and pharmacological functions, including anti-oxidant and anti-inflammatory properties, protected HT22 cells from oxygen-glucose deprivation and reoxygenation-induced injuries by reducing DNA damage (decreases in 8OHdG levels) [39]. In addition, we found that the immunoreactivity of 4HNE (an end-product of lipid peroxidation) in CA1-3 pyramidal cells was significantly increased on day 1 after IR. However, ATX significantly decreased 4HNE immunoreactivity. These results indicate that pretreatment with ATX mitigated severe IR-induced oxidative stress in CA1-3 pyramidal cells, which may be closely associated with ATX-mediated neuroprotection against severe IR injury.

The antioxidant defense system neutralizes or scavenges ROS and plays a protective role against ischemic brain injury [11,40]. One of the key enzymes in the antioxidant defense system is SOD, which acts first to defend against the harmful effects of oxidative stress. Targeting SOD expression has shown substantial success in animal models of transient brain ischemia. Sugawara et al. (2002) showed that IR-induced superoxide production and the selective death of CA1 pyramidal cells were significantly decreased in transgenic rats overexpressing SOD1 [41]. Keller et al. (1998) also found that lipid peroxidation, protein nitration, and brain infarction following transient focal cerebral ischemia were significantly reduced in transgenic mice overexpressing SOD2 [42]. Moreover, we reported that the increased expression of SOD1 and SOD2 in CA1 pyramidal cells following pretreatment with natural antioxidants derived from plant materials, such as quercetin and chlorogenic acid, contributed to the protection of CA1 pyramidal cells against IR injury in gerbils [43,44]. In this study, SOD1 and SOD2 immunoreactivity in the CA1-3 pyramidal cells of both the vehicle-IR and ATX-IR groups were gradually decreased with time after severe IR, but, in the ATX-IR group, SOD1 and SOD2 immunoreactivity were significantly high compared to the vehicle-IR group. These results suggest that SOD1 and SOD2 in CA1-3 pyramidal cells were increased by ATX pretreatment and these increases could contribute to the neuroprotection against oxidative stress following severe IR.

Collectively, this study showed that pretreatment with ATX attenuated the massive loss (death) of CA1-3 pyramidal cells in gerbil hippocampi following severe IR injury induced by 15-min transient forebrain ischemia, and the pretreatment with ATX significantly enhanced the expressions of SOD1 and 2 in the CA1-3 pyramidal cells before IR and reduced oxidative stress in pyramidal cells following severe IR. These findings suggest that ATX can be used as a potential dietary supplement to prevent the progression of severe IR injury in brains. However, we need an in-depth study on the mechanism of the neuroprotective effect of ATX. For instance, examination of changes in antioxidant markers such as nuclear factor erythroid 2-related factor 2 (Nrf2) should be conducted to demonstrate the antioxidant defense of ATX against IR. Moreover, since IR injury in gerbils triggers delayed neuronal death in the hippocampus, examination of behavioral changes needs to be carried out to demonstrate the amelioration of IR-induced cognitive decline by treatment with ATX.

## 4. Materials and Methods

### 4.1. Animals

The protocol of all experiments was approved (approval no., KW-2000113-1) on 13 January 2020 by the Ethics Committee of Kangwon National University (Chuncheon, Gangwon, Korea). Animal suffering during the whole experiments was minimized. Six-month male gerbils (*Meriones unguiculatus*), weighing 70–80 g, were used. The gerbils originated from Charles River Laboratories International, Inc. (Wilmington, MA, USA). The gerbils were housed in pathogen-free environment under standard condition at a controlled temperature (about 24 °C) and humidity (about 60%) on a 12:12 h light–dark cycle.

### 4.2. Groups and Treatment of ATX

The gerbils were blindly and randomly divided into four groups: (1) vehicle treated and sham operated group (sham group, *n* = 14), which was treated with vehicle (saline) and received sham IR; (2) vehicle-treated and IR-operated group (IR group, *n* = 21), which was treated with saline and received IR; (3) ATX-treated and sham-operated group (ATX-sham group, *n* = 14), which was treated with ATX and received sham; (4) ATX-IR group (*n* = 21), which was treated with ATX and received IR.

The chemical structure of ATX (SML0982; Sigma-Aldrich, St. Louis, MO, USA) and the plan schedule of the experiment are shown in Figure 1A. ATX (100 mg/kg) was dissolved in saline and injected intraperitoneally once a day for three consecutive days before IR. The dosage and duration of ATX treatment were selected based on a recent study showing that pretreatment with 100 mg/kg of ATX provided robust neuroprotection against transient focal cerebral ischemia-induced brain injury in rats [45].

The gerbils (*n* = 7, respectively) in the IR groups on day 1, day 2, and day 5 after IR, and the ones (*n* = 7, respectively) in the sham groups were sacrificed at 0 h and five days after IR to reduce the numbers

### 4.3. Induction of Severe IR Injury

Severe IR injury in the hippocampus was induced as follows. As described in a previous study [6], in brief, the gerbils were anesthetized with 2.5% isoflurane (4 L/min; JW Pharmaceutical Corporation, Seoul, Korea). Midline cervical incision was executed in the neck, and bilateral common carotid arteries, which supply arterial blood to the brain, were isolated from the carotid sheath and ligated with clips (Fine Science Tools, Foster City, CA, USA) for 15 min to develop severe IR injury in the forebrain, which contains the hippocampus. The complete stop of the blood supply was confirmed by the right and left central arteries located in the retinae using HEINE K180 ophthalmoscope (Heine Optotechnik, Herrsching, Germany). The clips were removed to restore the blood supply and the incision region was sutured. Body temperatures were controlled at normothermia (37 ± 0.5 °C) using a heating lamp (Harvard Apparatus, Holliston, MA, USA) and TR-100 rectal temperature probe (Fine Science Tools, Foster City, CA, USA). The gerbils of all sham groups received identical surgery without the ligation of the arteries. After the sham and IR operation, the gerbils were housed in adequate rooms (about 24 °C of temperature and about 55% of relative humidity).

### 4.4. Preparation of Histological Sections

Brain sections containing the hippocampus were prepared in the usual way. In brief, the gerbils were deeply anesthetized with urethane (1.5 g/kg, intraperitoneal injection). Under the deep anesthesia, the gerbils were rinsed by transcardial perfusion of 50 mM phosphate-buffered saline (pH 7.4) and fixed with 4% paraformaldehyde. Thereafter, the fixed brains were obtained from the skulls and more fixed in the 4% paraformaldehyde overnight and soaked in 25% sucrose for cryoprotection. Thereafter, coronal sections of 30-µm thickness were made using SM2010 R sliding microtome (Leica Biosystems, Wetzlar, Germany).

### 4.5. Nissl Staining

Nissl staining is a widely used method to study the morphology and pathology of neural tissue. To examine IR injury in the gerbil hippocampus, Nissl staining was performed in the usual way. Briefly, the sections were submerged in 0.1% CV acetate solution for 20 min at room temperature. These sections were briefly washed in distilled water, dehydrated in graded ethanol and then cleared in xylene. Lastly, they were mounted with Canada balsam (Sigma-Aldrich).

### 4.6. FJB Staining

FJB stains all degenerating neurons regardless of specific insult or mechanism of cell death. FJB staining was performed, as described previously [37]. In short, the sections were soaked in 0.06% potassium permanganate and incubated in 0.0004% FJB (Cell Signaling Technology, Beverly, MA, USA). Thereafter, these sections were washed and put on a hot plate (about 50 °C) for the reaction of FJB. Finally, the sections were cleared in xylene and coverslipped with DPX (Thermo Fisher Scientific, Waltham, MA, USA).

### 4.7. Immunohistochemistry

Immunohistochemistry was conducted in the usual way to evaluate neuronal damage using NeuN antibody, and to examine oxidative stress using 8OHdG antibody, 4HNE antibody, and endogenous antioxidant (SOD1 and SOD2) antibodies (Table 1). In brief, the sections were reacted with 0.3% hydrogen peroxide and subsequently immersed in 5% normal horse serum at room temperature. After washing, these sections were incubated in each primary antibody solution overnight at 4 °C. Subsequently, the sections were exposed to corresponding secondary antibody, as shown in Table 1, for two hours at room temperature and to avidin-biotin complex (diluted 1:200, Vector) for one hour at room temperature. These sections were then visualized using 0.06% 3,3′-diaminobenzidine tetrahydrochloride (Sigma-Aldrich Co., St. Louis, MO, USA) for one minute, and the immunoreactions were checked. Finally, they were mounted onto gelatin-coated slides and were sealed with mounting medium.

### 4.8. Analyses of Data

For quantitative analysis of IR-induced neuronal death, nine sections per animal were selected at the corresponding levels using the brain atlas of gerbil [46]. In short, as described previously [47], the digital images of NeuN- and FJB-stained cells were taken using a light microscope (BX53, Olympus, Tokyo, Japan) and an epifluorescent microscope (Carl Zeiss, Göttingen, Germany), respectively. The cell count was evaluated using Optimas 6.5 (an image analyzing system; CyberMetrics, Scottsdale, AZ, USA).

To quantitatively analyze the changes in 8OHdG, 4HNE, SOD1, and SOD2 immunoreactivities, a relative optical density (ROD) was applied. As described previously [48], in short, the digital image of immunostained structure was obtained using BX53 light microscope. The obtained image was converted into grey scale image (from black to white). The grey image was calculated by using Image J software (version 1.46) of the National Institutes of Health (Bethesda, Maryland, MD, USA).

### 4.9. Statistical Analyses

Statistical analyses were performed with aid of GraphPad Prism (version 5.0) (GraphPad Software, La Jolla, CA, USA). The differences of the means among the groups were statistically analyzed by two-way analysis of variance (ANOVA) tests with post hoc Bonferroni’s multiple comparison tests to elucidate IR-related differences among the groups. Data were presented as the means ± standard errors of the mean (SEM), and statistical significance was designated when *p* value was less than 0.05.

## Figures and Tables

**Table 1:** Primary and secondary antibodies for immunohistochemical staining.

Primary Antibodies	Dilutions	Suppliers
Mouse anti-neuronal nuclei (NeuN)	1:1000	Chemicon, Temecula, CA, USA
Goat anti-8-hydroxydeoxyguanosine (8OHdG)	1:500	Millipore, Billerica, MA, USA
Mouse 4-hydroxy-2-nonenal (4HNE)	1:800	Alexis Biochemicals, San Diego, CA, USA
Sheep anti-Cu, Zn-superoxide dismutase (SOD1)	1:800	Calbiochem, La Jolla, CA, USA
Sheep anti-Mn-superoxide dismutase (SOD2)	1:800	Calbiochem, La Jolla, CA, USA
**Secondary Antibodies**	**Dilutions**	**Suppliers**
Biotinylated horse anti-mouse IgG	1:250	Vector Laboratories Inc., Burlingame, CA, USA
Biotinylated horse anti-goat IgG	1:250	Vector Laboratories Inc., Burlingame, CA, USA
Biotinylated rabbit anti-sheep IgG	1:250	Vector Laboratories Inc., Burlingame, CA, USA

## Data Availability

The data presented in this study are available on request from the corresponding author.

## Abbreviations

4HNE	4-hydroxy-2-nonenal
8OHdG	8-hydroxydeoxyguanosine
ATX	astaxanthin
CA	cornu Ammonis
CV	cresyl violet
DG	dentate gyrus
FJB	Fluoro-Jade B
IR	ischemia and reperfusion
NeuN	neuronal nuclei
ROD	relative optical density
ROS	reactive oxygen species
SOD	superoxide dismutase
SO	stratum oriens
SP	stratum pyramidale
SR	stratum radiatum
